# Allergic bronchopulmonary aspergillosis (ABPA) in an atopic patient with difficult-to-expectorate airway secretions 

**DOI:** 10.5414/ALX02200E

**Published:** 2021-05-27

**Authors:** Marcus Joest

**Affiliations:** Lung and Allergy Center Bonn, Germany

**Keywords:** ABPA, allergic bronchopulmonary aspergillus, recombinant Aspergillus antibodies

## Abstract

In the workup of a 55-year-old atopic patient with cough and viscous secretions, we diagnosed an allergic bronchopulmonary aspergillosis (ABPA) on the basis of common diagnostic criteria for adult asthma patients (Rosenberg-Patterson and ISHAM), supported by the use of IgE antibodies against the Aspergillus components Asp f 2, f 4, and f 6. Initial treatment with prednisolone and itraconazole led to remission. In the long-term follow-up, there were few relapses until 2015, which responded well to standard treatment with oral steroids, and since 2016 the patient is in stable remission. The case highlights the valuable contribution of Aspergillus IgE measurements, including the specific IgEs against the components Asp f 1, f 2, f 4, and f 6 in the diagnosis of ABPA.

## Case report 

### Patient history 

A 55-year-old male patient in good general condition presented for the first time in July 2010. He described rhinitis that had been present for 2 months and cough symptoms with viscous secretions that were difficult to expectorate and were yellowish in color. In the previous few days, he had occasionally coughed up a “worm-like plug of secretion”. Occasionally, there was also discrete shortness of breath associated with the cough, so the family physician prescribed salbutamol DA as needed, but the patient used it very rarely. 

For many years, the patient had been known to have tree pollen allergy with seasonal rhinoconjunctivitis in March and April and oral allergy syndrome to pome fruits. However, the hay fever had improved in recent years. Otherwise, there were no relevant previous illnesses. 

The patient was a journalist, there was no obvious exposure to dust or molds in his professional or private life and no pets. 

### Findings 

Clinical examination on July 15, 2010, revealed a somewhat attenuated breath sound apically over both lungs with otherwise unremarkable findings. 

Pulmonary function revealed borderline obstructive ventilation disorder with normal airway volumes (vital capacity 5.3 L = 108%, FEV_1_ 3.5 L = 93%, FEV_1_/FVC (Tiffenau) = 66%, RAW_tot_ 0.29 kPa/L*s = 98%). 

Fractional NO in the exhalate (FeNO) was significantly elevated at 95 ppb (norm < 25 ppb). 

Skin tests revealed immediate reactions to the two molds *Aspergillus fumigatus* and *Alternaria alternata* as well as sensitization to birch, ash, and grass pollen and house dust mites (Table 1). 

The laboratory values (Table 2) showed a massively increased total IgE with significantly increased specific IgE antibodies against *Aspergillus fumigatus* (including its components) and *Alternaria alternata.* In addition, there was an elevated IgG against *Aspergillus fumigatus* in the sense of a type-3 reaction, as well as an eosinophilia. 

**Figure 1.**
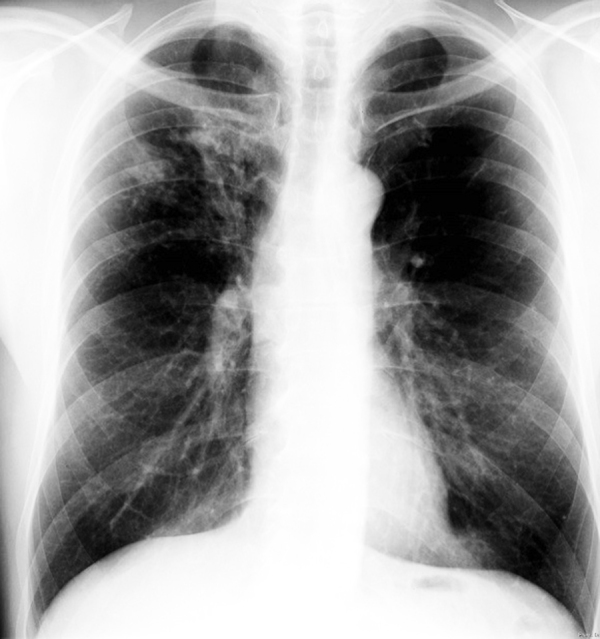
Chest x-ray in August 2010.

Chest X-ray on August 18, 2020 (Figure 1), showed focal patchy compressions in the right upper lobe and at the cardiac apex, no effusion. The hilar region was unremarkable. 

### Evaluation 

The strongly elevated total IgE as well as the elevated specific IgE antibodies against *Aspergillus fumigatus* and its components Asp 2, Asp f 4, and Asp f 6 raised the suspicion of the presence of allergic bronchopulmonary aspergillosis (ABPA), although the bronchial symptoms were mild except for the coughing up of secretory granules at initial presentation, and the pulmonary function showed only borderline obstruction with, however, a marked increase in FeNO, consistent with Th-2 bronchial asthma. 

Based on the subsequent diagnostic workup (differential blood count, specific *Aspergillus* IgG, chest X-ray), a diagnosis of ABPA was finally made. Five major and two minor criteria of the Rosenberg-Patterson diagnostic criteria were present (Table 3) [1]. The 2021 International Society for Human and Animal Mycology (ISHAM) criteria, last revised, were also met (Table 4) [2, 3]. 

The high-level sensitization to the mold *Alternaria* could also still direct suspicion to allergic bronchopulmonary mycosis (ABPM) caused by this fungus. Despite its ubiquitous occurrence, *Alternaria* triggers ABPM much less frequently than *Aspergillus fumigatus* and is also treated with systemic steroids in the same way as ABPA so that in the further course only the *Aspergillus* parameters were controlled. 

### Therapy 

Under systemic steroid therapy with initially 40 mg prednisolone daily, which could be phased out over 5 months, as well as itraconazole 200 mg daily during the first 4 weeks, the symptoms subsided rapidly, the radiograph normalized, and the serological parameters decreased (Table 5). 

### Course 

After a long stable course under asthma therapy using an inhaled steroid, relapses with clinical deterioration and significant increases in total IgE and specific *Aspergillus* IgE antibodies occurred again in October 2014 and August 2015 (Table 6). 

The relapses were ameliorated by several weeks of systemic steroid administration, and no further relapses occurred in the last 4 years with inhaled asthma therapy only. No bronchiectasis was found on a chest CT carried out in May 2016. 

## Context (literature review) 

ABPA is a complex clinical entity that usually develops on the background of allergic asthma or cystic fibrosis. The clinical picture can vary widely from severe disease to only mild symptoms, as in the case described, in which ABPA-S, i.e. ABPA without bronchiectasis and without permanent radiographic changes of the lungs, is present. If left untreated, irreversible changes such as bronchiectasis, fixed obstruction, pulmonary fibrosis, and respiratory failure may result. 

Serology plays a central role in ABPA diagnosis. Rosenberg and Patterson’s diagnostic criteria (Table 3), used since 1977, include an elevated total IgE of at least 1,000 ng/mL (equivalent to 418 kU/L today). In more recent literature, threshold values for total IgE of 500 kU/L or 1,000 kU/L are frequently used [2, 3, 16], although the value of 500 kU/L is to be preferred in screening due to its higher sensitivity. In addition, type 1 sensitization to *Aspergillus fumigatus* must be present, which can be detected by a positive skin test and a specific IgE > 0.35 kU/L, although much higher IgE concentrations are usually present. 

In the 1990s, the Davos working group first described the importance of IgE antibodies against specific *Aspergillus* epitopes, in particular Asp f 2, f 4, and f 6, for the diagnosis of ABPA in both asthma and cystic fibrosis patients [4, 5, 6, 7, 8]. Subsequently, other research groups have also confirmed that these three components have high specificity in the diagnosis of ABPA in European patients [9, 10]. A 2017 meta-analysis calculated a pooled specificity of 99.2% for Asp f 4 + Asp f 6 in diagnosing ABPA in (mostly European) asthmatics [11]. 

However, in recent years it has been shown that some *Aspergillus* components cross-react with other fungi [12], for example Asp f 6 with manganese superoxide dismutase (MnSOD) of *Malassezia sympodialis* [13]. In addition, the peroxisomal protein Asp f 3 also exhibits cross-reactions with Cand b 2 (*Candida*) and Mala f 3, 4 (*Malassezia*) [14]. 

A recent work from India has now shown that the combination of IgE antibodies against Asp f 1 (at a cutoff of 4.4 kU/L) and Asp f 2 (at a cutoff of 1.3 kU/L) have a sensitivity of 100% and a specificity of 81% in differentiating between *Aspergillus*-sensitized asthma patients and patients with ABPA in asthma [15]. 

For follow-up during therapy and for the detection of a relapse, the determination of total IgE is usually sufficient; here, antibodies against the *Aspergillus* components have only a limited additional benefit [16]. However, they are helpful for follow-up when other causes besides ABPA might have an influence on total IgE, such as atopic dermatitis, parasitosis, infection, or additional seasonal pollen allergy. 

For ABPA screening in asthma patients, in the future, based on the current data from India, the excellent combined sensitivity of Asp f 1 and Asp f 2 may allow these two parameters to be used in combination with total IgE instead of specific IgE against total *Aspergillus* extract. The determination of sIgE against Asp f 4 and f 6 can then be performed for confirmation due to the very good specificity of these two parameters. It would be desirable to verify the value of the two parameters Asp f 1 and f 2 in a study including European patients, since genetic differences of the patients as well as variations of the *Aspergillus* strains in India and Europe may well play a role [17]. 

## Conclusion 

ABPA may additionally be present even in mild or moderate asthma. Therefore, in any type of asthma and in cystic fibrosis, total IgE determination is useful to avoid overlooking ABPA. If total IgE values exceed 500 kU/L and there is simultaneous evidence of *Aspergillus* sensitization in the specific IgE or in the skin test, ABPA should then be considered. The IgE antibodies against the *Aspergillus* components Asp f 1, f 2, f 4, and f 6 make a valuable further contribution in the diagnosis of ABPA. 

## Acknowledgment 

I thank Prof. Dr. Joachim Sennekamp for critical review of the manuscript and his valuable comments. 

## Funding 

None. 

## Conflict of interest 

The author declares no conflict of interest. 

**Table 1.** Skin prick test (molds tested intracutaneously (i.c.)).

	Skin prick test	
Histamine	++	
NaCl	0	
Grass pollen	+	
Birch	+++	
Ash	++++	
Herb pollen	0	
*D. pteronyssinus*	+	
*D. farinae*	+ i.c.	Late reaction
*Alternaria alternata*	+++	++
*Aspergillus fumigatus*	+++	++

**Table 2.** Laboratory values on July 25, 2010.

Parameter		Normal range	Result	Semiquantitative score
Total IgE		< 100 kU/L	6,020 kU/L	
Specific IgE against	total Aspergillus extract	< 0.35 kU/L	78.5 kU/l	CAP class 5
	Asp f 2	< 0.35 kU/L	11.1 kU/L	CAP class 3
	Asp f 4	< 0.35 kU/L	0.53 kU/L	CAP class 1
	Asp f 6	< 0.35 kU/L	0.40 kU/L	CAP class 1
	*Alternaria alternata*	< 0.35 kU/L	100 kU/L	CAP class 6
Specific IgG against *Aspergillus fumigatus*		< 64 mgA/L	107 mgA/dL	Moderately elevated
Eosinophils in blood		< 4%/< 400/µL	6.5%/520/µL	

**Table 3.** Rosenberg-Patterson diagnostic criteria of ABPA [1].

**Main criteria**
Bronchial asthma
Positive immediate skin test reaction to *Aspergillus fumigatus*.
Total IgE > 417 IU/mL
Positive specific IgE against *Aspergillus fumigatus*
IgG antibodies against *Aspergillus fumigatus*
Blood eosinophilia (> 1,000 eos/µL)
Central bronchiectasis
Radiologically volatile or permanent pulmonary infiltrates
**Ancillary criteria**
Tough mucus plugs
Positive sputum culture for *Aspergillus fumigatus*
Late reaction in the intradermal test for *Aspergillus fumigatus*

**Table 4.** Modified ISHAM criteria 2021 [3].

**Criteria combination with best sensitivity/specificity**
Bronchial asthma
*Aspergillus fumigatus*-specific IgE > 0.35 kU/L
Total IgE > 500 IU/mL
**and at least two of the following criteria**
*Aspergillus fumigatus*-specific IgG > 27 mgA/dL
Bronchiectasis in thorax CT
Eosinophilia in blood > 500/µL

**Table 5.** Total IgE and specific IgE before and after therapy.

(kU/l)	July 15, 2010	September 15, 2010	November 8, 2010	May 10, 2012	February 25, 2013
Total IgE	6,020	4,954	2,740	808	638
*A. fumigatus*	78.5	56.2	30.6	20.8	
Asp f 2	11.1				
Asp f 4	0.53				
Asp f 6	0.40				

**Table 6.** Serological parameters in the further course.

(kU/L)	October 6, 2014	January 23, 2015	August 20, 2015	December 1, 2015	March 7, 2016
Total IgE	2.422	1.462	5.870	1.482	985
Specific IgE against					
*A. fumigatus*	46.2	32.0	56.8		34.1
Asp f 2	2.30	1.46	27.7		
Asp f 4	0.08		0.32		
Asp f 6	0.06		0.14
